# Development of a Simple Fabrication Method for Magnetic Micro Stir Bars and Induction of Rotational Motion in *Chlamydomonas reinhardtii*

**DOI:** 10.3390/mi13111842

**Published:** 2022-10-27

**Authors:** Ichiro Shimizu, Kyohei Yamashita, Eiji Tokunaga

**Affiliations:** Department of Physics, Faculty of Science, Tokyo University of Science, 1-3 Kagurazaka, Shinjuku-ku, Tokyo 162-8601, Japan

**Keywords:** magnetic micro stirrer bar, microchip, motion analysis, microalga, *Chlamydomonas*, neodymium magnet, flagella, orbital motion

## Abstract

A magnetic micro stirrer bar (MMSB) is used in the mixing operation of microfluidic devices. We have established a low-cost and easy method to make MMSBs using magnetic (neodymium magnets, magnet sheets) or non-magnetic powders (SUS304) as materials. We demonstrated three kinds of MMSB have respective advantages. To confirm the practical use of this MMSB, a cell suspension of the motile unicellular green alga *Chlamydomonas reinhardtii* was stirred in microwells. As a result, the number of rotating cells increased with only one of the two flagella mechanically removed by the shear force of the rotating bar, which facilitates the kinetic analysis of the flagellar motion of the cell. The rotational motion of the monoflagellate cell was modeled as translational (orbital) + spinning motion of a sphere in a viscous fluid and the driving force per flagellum was confirmed to be consistent with previous literature. Since the present method does not use genetic manipulations or chemicals to remove a flagellum, it is possible to obtain cells in a more naturally viable state quickly and easily than before. However, since the components eluted from the powder material harm the health of cells, it was suggested that MMSB coated with resin for long-term use would be suitable for more diverse applications.

## 1. Introduction

Efficient mixing of the reactants is beneficial to increase the reaction rate in chemical and biological reactions. Mechanical stirrers or commercially available magnetic stir bars are often used for efficient mixing in reactors of various sizes. However, mechanical stirrers or commercially available magnetic stir bars are impractical in micro-sized reactors, such as micro channels and droplets that are important in microfluidic devices [1], such as lab-on-a-chip. Therefore, it is desired to produce sufficiently small and low-cost stir bar for mixing. Furthermore, introduction and operation of the stir bar must be easy. Thus, in this study, we newly developed a micro-sized magnetic stir bar with simple introduction and operation. Usually, magnetic particles [2] are used in the fabrication of a magnetic micro stir bar, but it is necessary to purchase commercially available magnetic particles or to chemically fabricate magnetic particles. The fabrication method developed in this study allows for the fabrication of simple magnetic micro stirrer bars by using lower-cost materials than commercially available magnetic particles.

To date, magnetic stirring is still the most convenient means. In microfluidics study, many methods for improving mixing have been developed. For example, various magnetic micro stir bars (MMSBs), such as a star-shaped micro stirrer [3] made by soft lithography and a magnetic bar [4] made of cobalt-based magnetic alloy cut by laser micromachining have been reported. In addition, study on stir bar composed of magnetic particles has been reported (e.g., the dynamics of the rotation motion due to the magnetic particles arranged in a chain shape [5,6], and critical frequency depending on length of the chain [6,7], simulation of rotation motion [8], length of the chain depending on rotation speed [9], quantification of mixing [10,11], etc.). When the magnetic particles are exposed to an external magnetic field, they are arranged like a rod and function as a stir bar by rotating an external magnetic field [9,12]. Most magnetic particles [13] are currently on the market and offer several attractive possibilities in biomedicine. The size of the magnetic particles ranges from nano to micro size, and the magnetic particles are coated with a polymer resin, such as silica [14]. Moreover, typical magnetic particles are surrounded by a non-magnetic coating to selectively bind to the biomaterial of interest (e.g., a particular cell, protein, or DNA sequence) [1]. Magnetic particles can provide functionality, such as sample mixing and manipulation, detection, reaction, and separation in microfluidic system. Furthermore, the physical properties of magnetic particles, their control, and medical applications have been studied [13]. For example, magnetic particles have been widely applied to biomedical applications such as drug and gene delivery, MRI contrast agents, magnetic separation, hyperthermia, and artificial cilia [15,16,17,18]. In addition, the magnetic particles can have functions other than stirring, such as a catalyst [19,20], a color barcode [21], and microneedles [22]. 

In this paper, we fabricated the MMSB using powders produced when neodymium magnets, SUS304, or magnetic sheets are rubbed with an abrasive cloth. The MMSB can be prepared by using an inexpensive material and a simple method without using a reagent. This method can be applied to various magnetic materials. 

To verify the performance of the MMSB, a unicellular green alga *Chlamydomonas reinhardtii* was used as a sample. *C. reinhardtii* grows by photosynthesis, and swims like breaststroke with two flagella. The body is about 10 μm long and senses light at the eyespot and moves to the optimal light environment for photosynthesis. The average forward speed of *C. reinhardtii* is about 100–200 μm/s [23]. We have been researching single-cell motility and photohydrogen production associated with photosynthesis [24]. By developing a scan-free absorbance spectral imaging A(*x*, *y*, *λ*) method, it has enabled us to acquire local absorption spectra within a single cell during swimming and after hydrogen production [25]. In order to study cell movement and hydrogen production in a microreactor, the technique of stirring the culture medium in the microreactor is important for homogenization of substances. In this paper, we demonstrate that the fabricated MMSB can be used to stochastically remove only one flagellum of *C. reinhardtii*. Techniques used to remove the flagella will contribute to the research area of elucidating the structure [26], motility [27], and regeneration [28] of *Chlamydomonas* flagella. In order to remove flagella from wild-type biflagellate *C. reinhardtii*, various methods including chemical treatment and mechanical shearing with a homogenizer, pH shock, and extreme temperature are usually used [28]. The method of removing flagella using the fabricated MMSB is easier than the conventional method, using chemicals or homogenizer, extreme temperature. In addition, we analyzed the kinetics of the orbital motion of monoflagellate *C. reinhardtii*, in which only one of the two flagella has been removed. While the motility of monoflagellate *C. reinhardtii* has been analyzed in mutants with only one flagellum [27], this method allows the analysis of cell motility in a more natural state because monoflagellate cells can be easily obtained by mechanical stimulation without genetic manipulation or the use of chemicals.

## 2. Materials and Methods

### 2.1. Fabrication Method for MMSB

**Figure 1:**
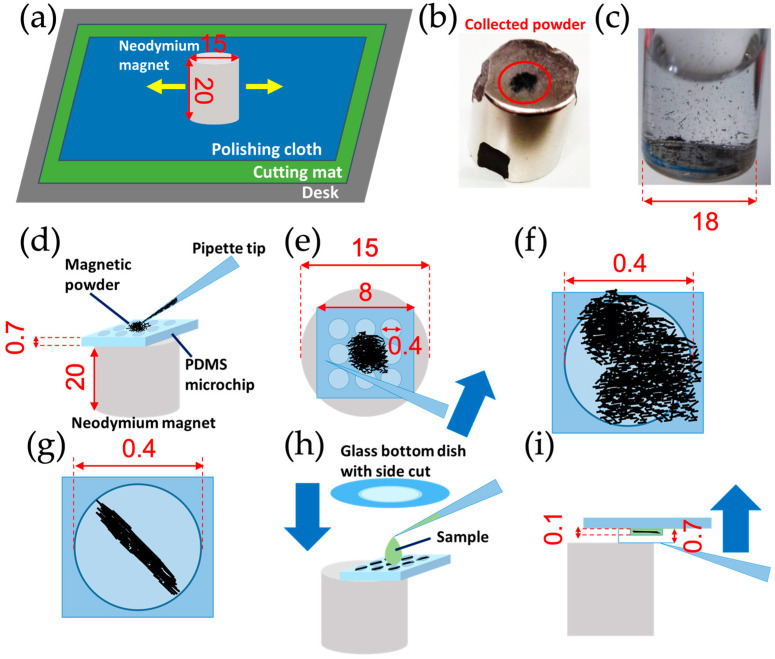
Schematic diagram of the fabrication process for the MMSB. All dimensions are in mm. (**a**) Place the cutting mat on the desk, place the polishing cloth on the cutting mat, and rub the neodymium magnet, SUS304, or the magnetic sheet. (**b**) Collect the powder adhering on the neodymium magnet in one place with latex gloved fingers. The part surrounded by red is the powder. (**c**) Pick up the powder collected with latex gloved fingers and disperse the powder in alcohol. (**d**) Put the PDMS microwell on the neodymium magnet. The alcohol dispersed with the powder is sucked up with a micro-pipette and injected onto the PDMS microchip. (**e**) Spread the powder with the pipette tip to put the powder into the microwell. (**f**,**g**) If there is too much powder in the microwell, flush the excess powder with alcohol while holding the PDMS microchip against the neodymium magnet. (**h**) Bring the PDMS microchip to the end of the neodymium magnet. Put cell suspension (*C. reinhardtii* suspension) on the PDMS microwell. Then press a glass bottom dish with only the bottom cut out over the PDMS microchip. (**i**) Lift the PDMS microchip and the glass bottom dish with the pipette tip.

The following work was performed while wearing latex gloves (Figure 1). Figure 1a shows the fabrication process of the MMSB. Neodymium magnets (ϕ1.5 cm × 2.0 cm, 564 mT, purchased from NeoMag Co., Ltd., Tokyo, Japan, Figure 1b), SUS304, and magnetic sheets (purchased from Daiso Industries Co., Ltd., Hiroshima, Japan), which are the materials of the stirrer bar, are rubbed against the polishing cloth (Mr. Lapros #2400, GSI Creos Corp., Tokyo, Japan), and pulverized. Since the powder adheres to the surface of the materials and the polishing cloth, the powder is attracted to a neodymium magnet for powder recovery. The powder adhering to the neodymium magnet is then collected in one place (Figure 1b), picked up with the latex-gloved fingers, and dispersed in 20 mL alcohol which consists of 88.40% ethanol, 10.49% isopropyl alcohol, and 1.11% methyl ethyl ketone (Imazu Chemical Co. Ltd., Tokyo, Japan) (Figure 1c). To wash the powder, stir the powder with a magnetic stirrer for about 1 min. After that, wash the powder about 3 times with alcohol and redisperse the powder in alcohol of 20 mL. A PDMS microchip is placed on the neodymium magnet and the dispersion is sucked up with a micro-pipette to dispense the dispersion into the PDMS microwell (*ϕ*400 µm, depth 100 µm: Figure 1d) on the microchip. In order to put the prepared powder into the PDMS microwell, spread the powder with the micro-pipette tip (Figure 1e). If there is too much powder in PDMS microwell, push out vigorously the alcohol in a wash bottle and flush the excess powder (Figure 1f,g). After the alcohol dries, bring the PDMS microchip close to the end of the neodymium magnet, place the cell suspension on the PDMS microchip, and cover the PDMS microchip with a glass bottom dish (D11130H, Matsunami Glass Ind., Ltd., Osaka, Japan) with a side cut into a disc shape with a plastic cutter (Figure 1h). After enclosing the cell suspension in the PDMS microwell, hold the glass bottom dish with fingers and lift the microchip and glass bottom dish with a pipette tip from below (Figure 1i). Lightly press the PDMS microchip against the glass bottom dish and absorb the cell suspension that oozed out with a Kimwipe. 

### 2.2. Observation Method for MMSB

**Figure 2:**
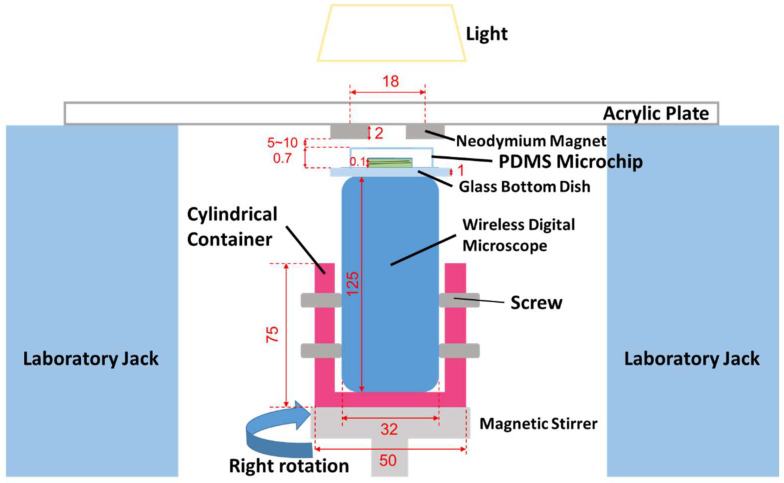
Schematic diagram of the observation system for the MMSB. All dimensions are in mm.

**Figure 3:**
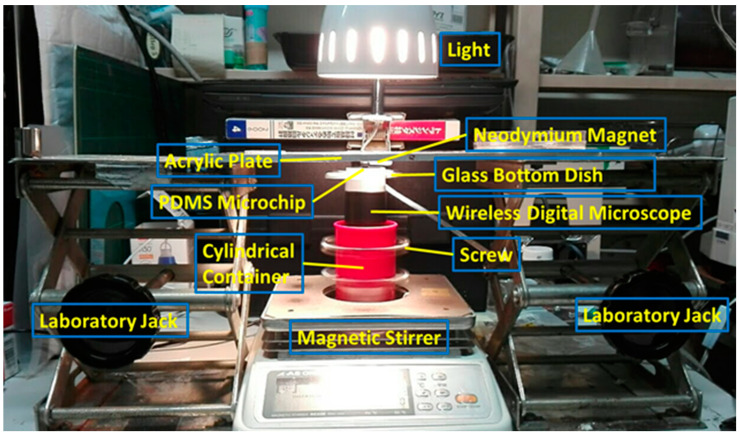
Photograph of the actual observation system for the MMSB.

**Figure 4:**
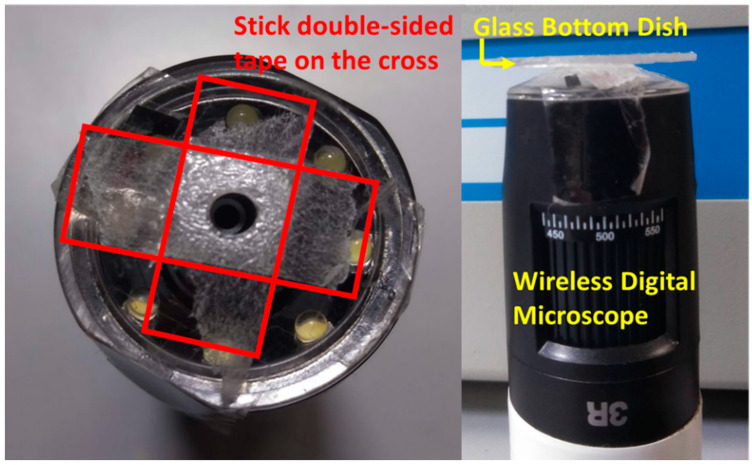
Glass bottom dish and microscope setup.

**Figure 5:**
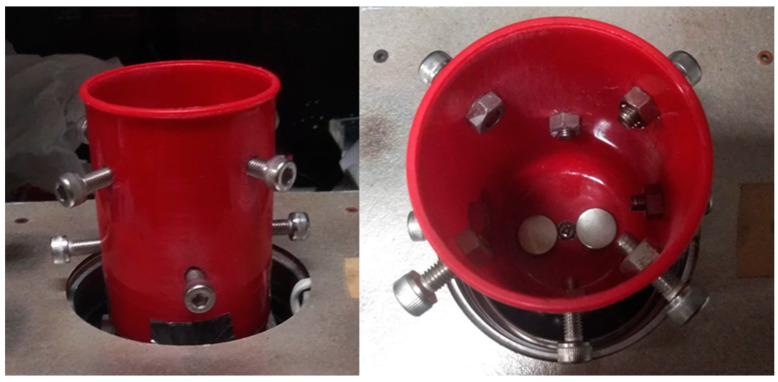
Cylindrical container with screws.

**Figure 6:**
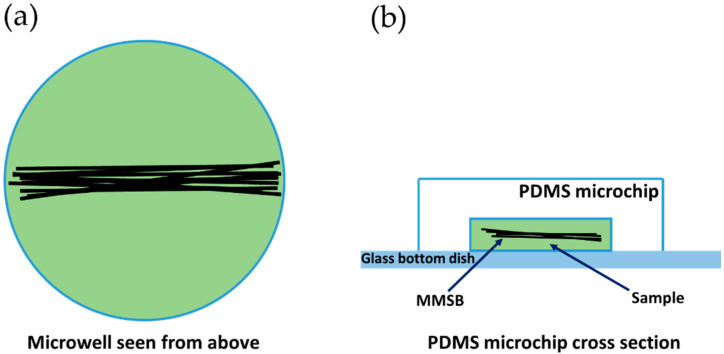
Schematic diagram after applying the external rotating magnetic field to the powder enclosed in the PDMS microwell. (**a**) A view of the PDMS microwell from directly above. (**b**) A view of the PDMS microwell from the side.

The MMSB was observed using a wireless digital microscope (500×, 3R-WM601PC, 3R SYSTEMS CORP.). Figure 2 shows a schematic diagram of the observation system. Figure 3 is the actual photograph of Figure 2. First, stick the double-sided tape on the release paper, and make a hole in the double-sided tape with a punch. Prepare two double-sided tapes with punched holes and stick them crosswise on the attached 500× lens cover (Figure 4). Then, attach the lens cover with the double-sided tapes to the wireless digital microscope. Next, fix the glass bottom dish containing the sample on the lens cover with the double-sided tape (Figure 4). After this, the wireless digital microscope was fixed to the cylindrical container with screws (Figure 5). The contact area between the screw and the microscope barrel was wrapped with vinyl tape to prevent slippage and protect the housing. The container and the rotating part of the magnetic stirrer (REXIM RSH-1DN, AS ONE) were fixed with neodymium magnets and aluminum tape. A colorless and transparent acrylic plate with two neodymium magnets attached (*ϕ*1.3 cm × 0.2 cm, 240 mT, purchased from Daiso Industries Co., Ltd., Hiroshima, Japan) was placed directly above the sample. The distance between the sample (PDMS microtip) and two neodymium magnets was adjusted to about 5–10 mm by a laboratory jack (Figure 2). An observation light source was placed above it (Figure 3). When the magnetic stirrer was rotated, the enclosed powder rotates synchronously. At this time, the randomly dispersed powder formed a rod shape and rotated. In the inertial coordinate system (laboratory frame), the microscope was rotating while the MMSB and the magnetic field were stationary. When viewed from the microscope, the MMSB and magnetic field were rotating. Figure 6 shows a schematic diagram after applying the external rotating magnetic field to the powder enclosed in PDMS microwell. The size of the MMSB depended on the diameter of the PDMS microwell and the size of the powder particles.

### 2.3. Sample Preparation

The wild type *C. reinhardtii* (NIES-2238) was cultured in TAP (tris–acetate–phosphate) medium for 3 days. Cells were kept aerobically under continuous illumination (cool white fluorescent light: 40 μmol/m^2^/s) and constant temperature (26 °C).

### 2.4. Effects of MMSB on C. reinhardtii

Effects of three types of MMSB materials (neodymium magnet, SUS304, magnet sheet) on cells were investigated. Cell suspensions were encapsulated in microchips together with MMSB and illuminated constantly with white fluorescent light. For each material, samples were illuminated with the following four types of light intensity, and cell motility was observed under a microscope.

Intense light (700 μmol m^−2^ s^−1^), Medium light (100 μmol m^−2^ s^−1^), Weak light (5 μmol m^−2^ s^−1^), Weaker light.

### 2.5. Observation of the Rotational Movement of C. reinhardtii

A cell suspension encapsulated in PDMS microwells was stirred at 120 RPM for 1 min with MMSB, and the rotational movement of *C. reinhardtii* with a single flagellum was observed. For this observation, a high-speed camera (FASTCAM Mini AX, Photron, Tokyo, Japan) and an inverted research microscope (IX71, OLYMPUS, Tokyo, Japan) equipped with a phase-contrast unit and an oil-immersion phase-contrast objective (×100, NA1.30, UPLFLN100XO2, OLYMPUS) was used. The photon flux density (PFD) at the sample installation position was 246 μmol m^−2^ s^−1^. Each test was performed with a MMBS of three different materials.

### 2.6. Trajectory Analysis of C. reinhardtii after Stirring

*C. reinhardtii* suspensions were stirred at 120 RPM for 1 min using MMBS made from magnet sheet powder. Video recordings of the cells before and after agitation were binarized with ImageJ [https://fiji.sc (accessed on 25 March 2020)], and the position of the center of each cell was tracked by its plug-in, TrackMate.

## 3. Results

**Figure 7:**
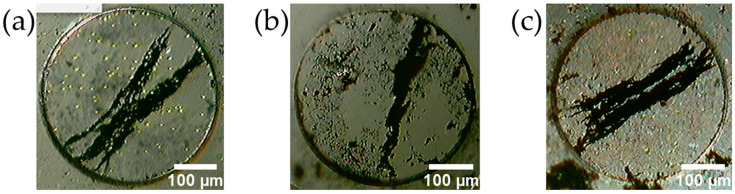
MMSBs in PDMS microwells (ϕ400 µm, depth 100 µm). (**a**) MMSB made of neodymium magnet. (**b**) MMSB made of SUS304. (**c**) MMSB made of magnet sheet.

**Figure 8:**
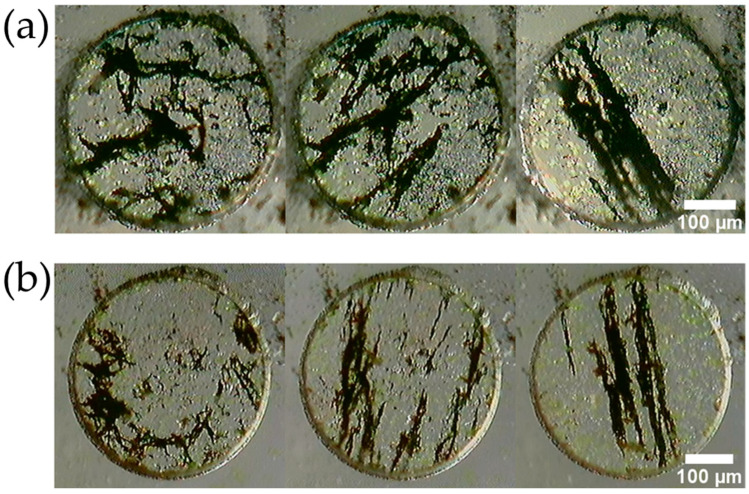
Shape change of the fabricated powder. (**a**) Powder of neodymium magnet. (**b**) Powder of magnet sheet.

Figure 7 shows the fabricated MMSBs. Figure 8 shows the forming process of the MMSBs. The left is immediately after the powder was enclosed in the PDMS microwell. The center is when the powder was brought close to the neodymium magnet in Figure 2. The right is after the magnetic stirrer, which was rotated at 120 RPM, was stopped. All PDMS microwells have a diameter of 400 μm and a well depth of 100 μm.

Table 1 shows the stability of bar shape during rotation, magnetism, and cytotoxicity for MMSB materials.

Table 2 shows the effect of MMSB material and PFD on cell motility.

**Figure 9:**
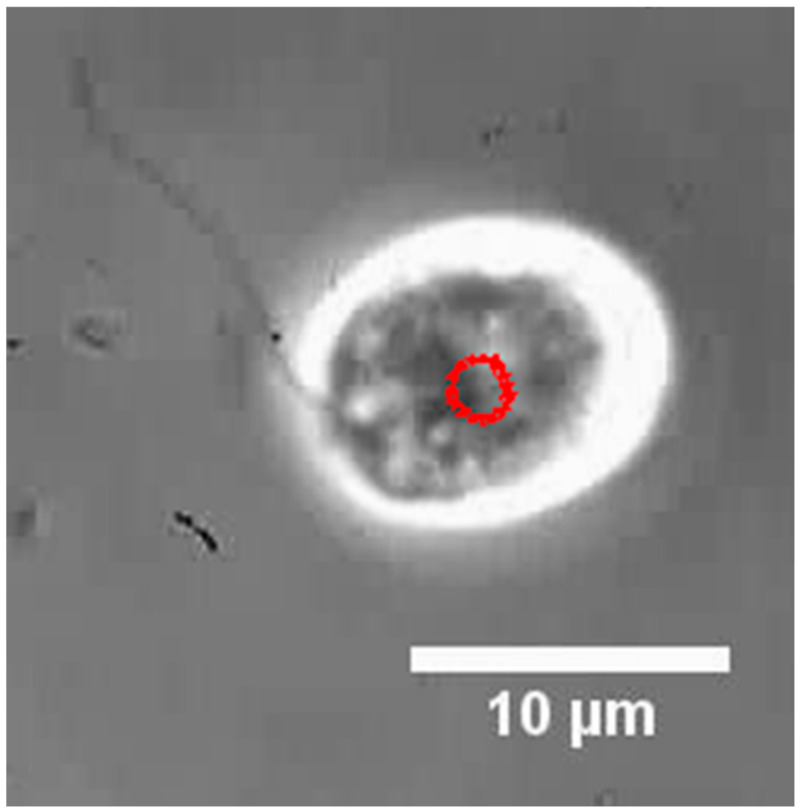
*C. reinhardtii* with only one flagellum removed.

Figure 9 shows a cell with only one flagellum removed and its orbital locus (red dots).

Red dots are the trajectory of the revolution motion. See the Supplementary Video S1.

**Figure 10:**
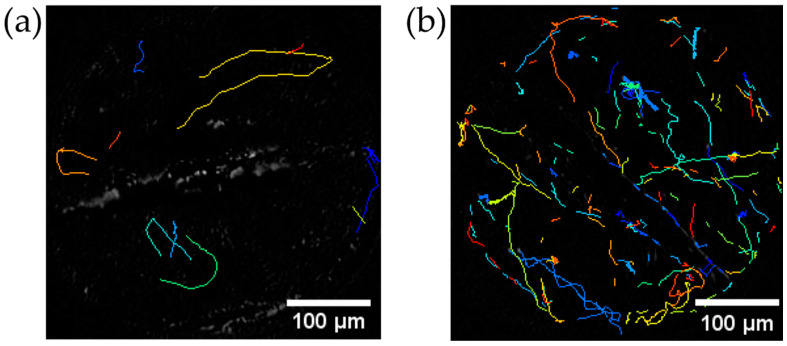
Trajectories of cell centers in *C. reinhardtii* for 11 s. (**a**) before stirring (**b**) immediately after 11-s stirring. Each color of the trajectory corresponds to each cell. The scale bars designate 5 µm.

Figure 10 shows the trajectory of the center of *C. reinhardtii*.

**Figure 11:**
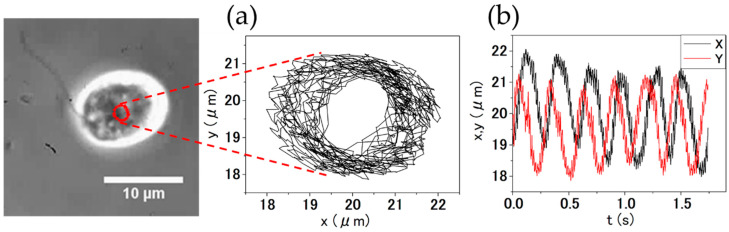
Revolution motion analysis of *C. reinhardtii* with only one flagellum. (**a**) XY plot of the revolution motion of *C. reinhardtii*. (**b**) Displacement of the center of revolving *C. reinhardtii*. Black represents the X coordinate of the center of *C. reinhardtii*, and red represents the Y coordinate of the center.

Figure 11a shows the XY plot of Figure 9. Figure 11b shows the displacement of the center of *C. reinhardtii* in orbital (revolution) motion.

**Figure 12:**
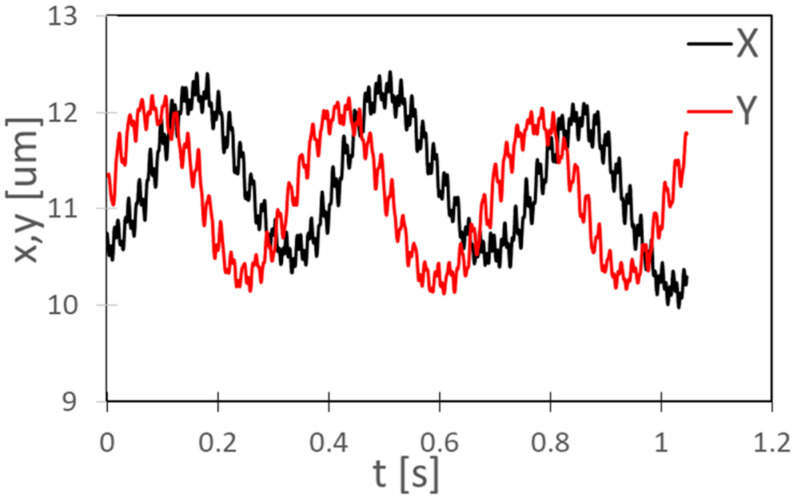
Displacement of the center of a *C. reinhardtii* during three rotations.

Figure 12 shows the displacement of the center of a *C. reinhardtii* during three revolutions. The beating frequency of the flagellum is 49 Hz, in agreement with the previous observations [29,30].

Similarly, the orbital radius and angular velocity were obtained from the displacement graph of the center of multiple *C. reinhardtii*. The results are shown in Table 3.

**Figure 13:**
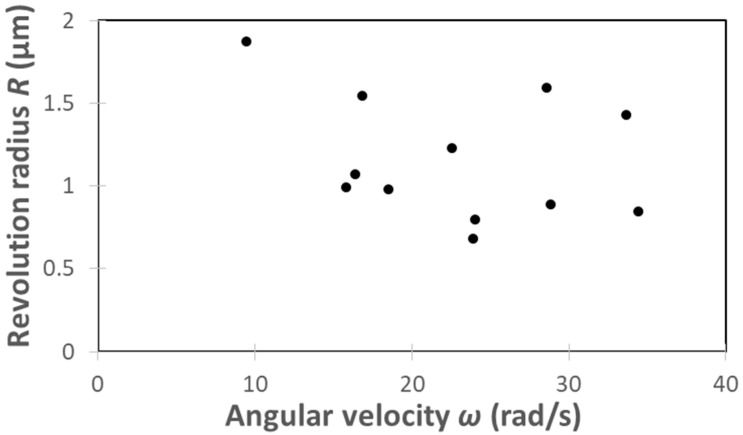
Distribution of angular velocity and revolution radius.

Figure 13 shows the distribution of the revolution radius and angular velocity in Table 3. The revolution radius and angular velocity were obtained by taking these average values. The revolution radius was calculated to be *R* = 1.16 × 10^−6^ m and the angular velocity was *ω* = 22.75 rad/s.

## 4. Discussion

### 4.1. Characteristics of the Fabricated MMSB

A micro-sized magnetic stirrer bar was fabricated using the powder generated when a neodymium magnet, SUS304, and a magnet sheet were rubbed with a polishing cloth (Figure 7). When the powder encapsulated in the PDMS microwells was placed in an external rotating magnetic field at 120 RPM by a magnetic stirrer, the dispersed powder formed into a rod shape and rotated while maintaining its shape (Figure 8). In the conventional method for making the MMSB, it is necessary to prepare commercially available magnetic particles [2] or polymer coated magnetic particles [13,14,31,32,33]. In this method, neodymium magnets, SUS304, magnet sheets, and polishing cloths can be used to easily and inexpensively produce magnetic micro stirrer bars.

It was found that the MMSB made of neodymium magnet or magnet sheet can be used without distortion of the stir bar shape even at 3000 RPM. The MMSB made of SUS304 did not maintain the shape of the stir bar at 3000 RPM and small lumps of powders rotated. SUS304 is non-magnetic, but when subjected to plastic deformation, such as friction, it undergoes a structural change and becomes weakly magnetic [34]. This is likely why the above results were obtained. The stronger the bonding force among the particles, the more the MMSB can withstand high-speed rotation. It was found that the MMSB made of neodymium magnet powder rotates most stably when stirring at high speed.

### 4.2. Effects of MMSB on C. reinhardtii

Since excessive iron and intense light synergistically have an adverse influence on the survival of *C. reinhardtii* [35,36,37], the effects of MMSB and PFD on the motility of cells were examined.

With the MMSB made of neodymium magnet, cells stopped motion in about 10 min regardless of PFD. With the MMSB made of SUS304, cells were weakened under intense light with a PFD of 700 μmol m^−2^ s^−1^. Or in another cell suspension, the motility of cells did not decrease. Under weak light with a PFD of 5 μmol m^−2^ s^−1^, cells were weakened over time. When the cell suspension was exposed under a lower PFD, the motility of cells recovered. In the MMSB made of magnet sheet, the cells were not weakened under medium light with a PFD of 100 μmol m^−2^ s^−1^ and survived actively for a couple of days. A typical MMSB is composed of magnetic particles and the particles are coated with a polymer such as silica [12,13,38]. A magnet sheet is made by kneading magnet (iron oxide) powder into polypropylene resin and processing it into a sheet. Powder produced by rubbing the magnet sheet with the polishing cloth has lower exposure ratio of iron oxide than neodymium magnet and SUS304 powder. From the above, it is suggested that the toxic effect on cells is small because the amount of iron dissolved in the cell suspension is small. Therefore, improvement is expected by coating MMSB with polymer [12,14,20].

### 4.3. Orbital Motion of C. reinhardtii

When the cell suspension was stirred with MMBS in the microwells, the number of rotating cells increased. This suggested that one flagella of *C. reinhardtii* was stochastically removed by the shear force caused by the rotation of the MMBS. As a result of plotting the center of cells with one flagellum over time, it was found that the center of the cell undergoes orbital movement. Figure 9 shows a cell with only one flagellum removed and its orbital locus (red dots). In long-term observation, cells with only one flagellum removed stopped orbiting. After some cells stopped moving, the flagella were rounded and detached. The stopped cells did not start moving again. It was suggested that this was caused by the iron eluted from MMBS, suggesting that this method is suitable for short-term experiments.

### 4.4. Trajectory of C. reinhardtii before and after Stirring

Figure 10 shows the trajectory of the center of *C. reinhardtii*. After 11 s of stirring (Figure 10b), the number of moving cells increased compared to 11 s before stirring (Figure 10a).

### 4.5. Kinematic Analysis of the Rotational Motion of C. reinhardtii

**Figure 14:**
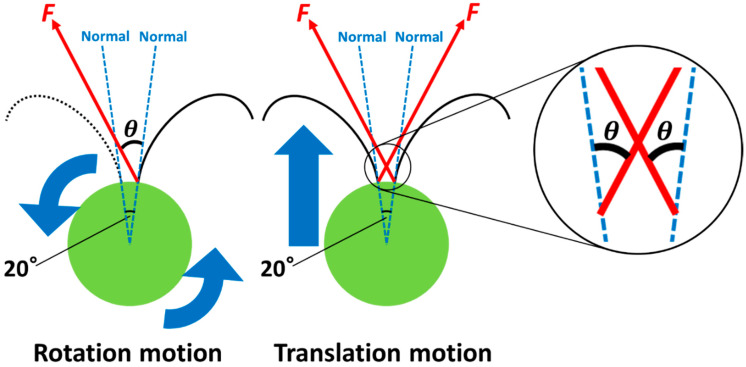
Schematic diagram of the translation and rotation motion of *C. reinhardtii*.

The movement of *C. reinhardtii* is modeled as shown in Figure 14. The cells are assumed to be spherical, with flagella growing at a central angle of 20° on the equator. The central angle of 20° was obtained from a microscope image of cells. We define the magnitude *F* of the driving force per flagellum averaged for the beating motion and the angle *θ* formed by the driving force *F* and the normal to the tangential plane of the cell (sphere) at the base of the flagella. In the analysis, first, *F* and *θ* are obtained from the equations for the translation (revolution) and rotation (spinning) of monoflagellate cells. In the moving cells, the power due to the flagellar driving force balances the energy dissipation rate due to the viscous resistance. Therefore, from the energy balance of the cell with one flagellum, the translation motion yields revolution (orbital) motion, and the rotation motion yields spinning motion. Next, the calculated *F* and *θ* are substituted into the expression for the translational motion of *C. reinhardtii* with two flagella, and the translational velocity *v* is obtained.

In Figure 14, assuming that the tangent direction of *F* yields spinning (rotation) motion and the normal direction yields revolution (translation) motion, the energy balance is expressed by the following equation.

(1) $$F\cos\theta R\omega +F\sin\theta r\omega =6\pi \eta r{\left(R\omega \right)}^{2}+8\pi \eta r^{3}\omega^{2},$$

where *R* is the revolution radius, *ω* is the angular velocity, *r* is the radius of the cell, and *η* is the viscosity of water. The viscous force and torque of the cell body are obtained from Stokes’ law [39,40], and the Equation (1) holds.

The revolution (translation) and rotation (spinning) motions of the cell body are then

(2) Fcosθ=6πηrRω,

(3) $$rF\sin\theta =8\pi \eta r^{3}\omega .$$

Calculations of Equations (2) and (3) yield, respectively.

(4) $$F\cos\theta =2.5\times 10^{-12}\;N$$

and

(5) $$F\sin\theta =14.3\times 10^{-12}\;N,$$

assuming that the radius of the cell is $r=5\times 10^{-6}$ m and the viscosity of water is $\eta =1.002\times 10^{-3}$ Pa·s. Moreover, the revolution radius is $R=1.16\times 10^{-6}$ m and the angular velocity is ω=22.75  rad/s by video image analysis.

The angle *θ* between the driving force *F* and the normal line at the position where the flagella are growing is obtained from

(6) $$\tan\theta =\frac{F\sin\theta }{F\cos\theta }=\frac{4r}{3R}=5.7$$

to be

(7) $$\theta =\tan^{-1}\theta =80.1{}^{\circ}.$$

The driving force F per flagellum is obtained from

(8) $$F^{2}={\left(F\sin\theta \right)}^{2}+{\left(F\cos\theta \right)}^{2}=2492.2\times 10^{-30}$$

to be

(9) $$F=14.5\times 10^{-12}\;N.$$

From Figure 14, the equation for the translation motion of the biflagellate cell is

(10) $$2F\cos\left(\theta -10^{\circ }\right)=6\pi \eta rv,$$

where *v* is the translation velocity of the biflagellate cell.

Since substituting *F*, *θ*, *η*, and *r* yields

(11) $$F\cos\left(\theta -10^{\circ }\right)=5.0\times 10^{-12}\;N$$

and

(12) $$3\pi \eta r\;=\;47.22\times 10^{-9}\;\mathrm{kg}/s$$

the translation velocity *v* of the biflagellate cells is obtained as

(13) $$v\;=\;\frac{F\cos\left(\theta -10^{\circ }\right)}{3\pi \eta r}\;=\;105\times 10^{-6}\;m/s.$$

This almost coincides with the average forward swimming speed of the cell [23,41]. As a matter of course, the present estimate for the average propulsion force of the biflagellate cell, $2F\cos\left(\theta -10^{\circ }\right)=10\;\mathrm{pN}$, is consistent with the previous estimate from the swimming speed in the literature [42]. Note also that the simple sum of F (2F=29 pN) agrees with the maximum force estimated by the direct force measurements [30,43].

## 5. Conclusions

Magnetic powders obtained from neodymium magnets, SUS304, and magnet sheets rubbed with a polishing cloth were enclosed in microwells, and a rotating magnetic field was applied from the outside to fabricate MMSBs by a simpler and more inexpensive method than before. When the *C. reinhardtii* cell suspension was stirred with MMSB, the stochastic removal of only one of the flagella increased the number of rotating cells. From the trajectory of the motion, it was confirmed that the rotating cells were in orbital motion, and the driving force per flagellum was calculated and found to be equivalent to the value in the prior literature. Since the preparation of monoflagellate cells by this method does not use genetic manipulations or reagents, it enables us to obtain monoflagellate cells in a more natural state. However, long-term coexistence with MMSB may harm cell health due to components eluted from the magnetic powder. Among the three types of materials, MMSB made of magnetic sheet had the lowest cytotoxicity. This is thought to be because the magnet (iron oxide) is coated with resin, so that elution from the magnet is suppressed. Therefore, by coating the MMSB with a material that prevents elution from the magnetic powder, it is expected that it will be used for more general purpose by eliminating the outflow of harmful components from the magnetic powder. 

## Figures and Tables

**Table 1:** MMSB of material type and properties.

Material of MMSB	Stability of Bar Shape	Magnetism	Cytotoxicity
Neodymium magnet	Stable	Very strong	High
SUS304	Unstable	Very weak	Medium
Magnet sheet	Stable	Strong	Low

**Table 2:** Effect of MMSB on cells.

Material of MMSB	Effects on the Cells
Neodymium magnet	Under weak light with a PFD of 5 μmol m^−2^ s^−1^, cells stopped moving in 10 min.
SUS304	Under weak light with a PFD of 5 μmol m^−2^ s^−1^, cells weakened over time (over 10 min). Under a lower PFD, motility recovered.
Magnet sheet	Under medium light with a PFD of 100 μmol m^−2^ s^−1^, the cells survived actively for several days.

**Table 3:** Angular velocity and revolution radius of *C. reinhardtii* obtained from video image analysis.

*C. reinhardtii*	Angular Velocity ω (rad/s)	Revolution Radius *R* (μm)
①	28.81	0.89
②	34.42	0.84
③	24.05	0.80
④	33.66	1.43
⑤	23.91	0.68
⑥	18.52	0.98
⑦	28.57	1.59
⑧	16.87	1.55
⑨	9.48	1.87
⑩	16.36	1.07
⑪	15.81	0.99
⑫	22.54	1.23

## Data Availability

The data presented in this study are available on request from the corresponding author.

## Supplementary Materials

The following supporting information can be downloaded at: https://www.mdpi.com/article/10.3390/mi13111842/s1, Video S1: The movie showing the trajectory of the revolution motion of the rotating monoflagellate cell in Figure 9. Recorded at 3000 fps and played back at 25 fps.

## References

[1] Xiong Q., Lim C.Y., Ren J., Zhou J., Pu K., Chan-Park M.B., Mao H., Lam Y.C., Duan H. (2018). Magnetic Nanochain Integrated Microfluidic Biochips. Nat. Commun..

[2] van Reenen A., de Jong A.M., den Toonder J.M.J., Prins M.W.J. (2014). Integrated Lab-on-Chip Biosensing Systems Based on Magnetic Particle Actuation—A Comprehensive Review. Lab Chip.

[3] Zhang Y., Wang T.-H. (2012). Micro Magnetic Gyromixer for Speeding up Reactions in Droplets. Microfluid. Nanofluid..

[4] Bruyker D.D., Recht M.I., Bhagat A.A.S., Torres F.E., Bell A.G., Bruce R.H. (2011). Rapid Mixing of Sub-Microlitre Drops by Magnetic Micro-Stirring. Lab Chip.

[5] Kang T.G., Hulsen M.A., Anderson P.D., den Toonder J.M.J., Meijer H.E.H. (2007). Chaotic Mixing Induced by a Magnetic Chain in a Rotating Magnetic Field. Phys. Rev. E.

[6] Vázquez-Quesada A., Franke T., Ellero M. (2017). Theory and Simulation of the Dynamics, Deformation, and Breakup of a Chain of Superparamagnetic Beads under a Rotating Magnetic Field. Phys. Fluids.

[7] Franke T., Schmid L., Weitz D.A., Wixforth A. (2009). Magneto-Mechanical Mixing and Manipulation of Picoliter Volumes in Vesicles. Lab Chip.

[8] Gao Y., Hulsen M.A., Kang T.G., den Toonder J.M.J. (2012). Numerical and Experimental Study of a Rotating Magnetic Particle Chain in a Viscous Fluid. Phys. Rev. E.

[9] Vuppu A.K., Garcia A.A., Hayes M.A. (2003). Video Microscopy of Dynamically Aggregated Paramagnetic Particle Chains in an Applied Rotating Magnetic Field. Langmuir.

[10] Roy T., Sinha A., Chakraborty S., Ganguly R., Puri I.K. (2009). Magnetic Microsphere-Based Mixers for Microdroplets. Phys. Fluids.

[11] Chang M., Gabayno J.L.F., Ye R., Huang K.-W., Chang Y.-J. (2017). Mixing Efficiency Enhancing in Micromixer by Controlled Magnetic Stirring of Fe_3_O_4_ Nanomaterial. Microsyst. Technol..

[12] Chong W.H., Chin L.K., Tan R.L.S., Wang H., Liu A.Q., Chen H. (2013). Stirring in Suspension: Nanometer-Sized Magnetic Stir Bars. Angew. Chem. Int. Ed..

[13] Ruffert C., Ruffert C. (2016). Magnetic Bead—Magic Bullet. Micromachines.

[14] Hu Y., He L., Yin Y. (2011). Magnetically Responsive Photonic Nanochains. Angew. Chem. Int. Ed..

[15] Pankhurst Q.A., Connolly J., Jones S.K., Dobson J. (2003). Applications of Magnetic Nanoparticles in Biomedicine. J. Phys. D Appl. Phys..

[16] Gijs M.A.M. (2004). Magnetic Bead Handling on-Chip: New Opportunities for Analytical Applications. Microfluid. Nanofluid.

[17] Peyer K.E., Zhang L., Nelson B.J. (2013). Bio-Inspired Magnetic Swimming Microrobots for Biomedical Applications. Nanoscale.

[18] Vilfan M., Potočnik A., Kavčič B., Osterman N., Poberaj I., Vilfan A., Babič D. (2010). Self-Assembled Artificial Cilia. Proc. Natl. Acad. Sci. USA.

[19] Miao L., Zhu Y.-Z., Wang H.-F. (2017). Nickel-Decorated Fe_3_O_4_ Nanoparticles as Recyclable Magnetic Self-Stirring Nanocatalysts for Microreactions. ACS Sustain. Chem. Eng..

[20] Wang S., Fu J., Wang K., Gao M., Wang X., Wang Z., Chen J., Xu Q. (2018). Facile Synthesis of Pd Nanoparticles on Polydopamine-Coated Fe-Fe_2_O_3_ Magnetic Nanochains as Recyclable High-Performance Nanocatalysts. Appl. Surf. Sci..

[21] Lee H., Kim J., Kim H., Kim J., Kwon S. (2010). Colour-Barcoded Magnetic Microparticles for Multiplexed Bioassays. Nat. Mater..

[22] Kavaldzhiev M., Perez J.E., Ivanov Y., Bertoncini A., Liberale C., Kosel J. (2017). Biocompatible 3D Printed Magnetic Micro Needles. Biomed. Phys. Eng. Express.

[23] Harris E.H. (2009). The Chlamydomonas Sourcebook.

[24] Yagi T., Yamashita K., Okada N., Isono T., Momose D., Mineki S., Tokunaga E. (2016). Hydrogen Photoproduction in Green Algae *Chlamydomonas*
*reinhardtii* Sustainable over 2 Weeks with the Original Cell Culture without Supply of Fresh Cells nor Exchange of the whole Culture Medium. J. Plant Res..

[25] Isono T., Yamashita K., Momose D., Kobayashi H., Kitamura M., Nishiyama Y., Hosoya T., Kanda H., Kudo A., Okada N. (2015). Scan-Free Absorbance Spectral Imaging A(*x*, *y*, *λ*) of Single Live Algal Cells for Quantifying Absorbance of Cell Suspensions. PLoS ONE.

[26] Barber C.F., Heuser T., Carbajal-González B.I., Botchkarev V.V., Nicastro D. (2012). Three-Dimensional Structure of the Radial Spokes Reveals Heterogeneity and Interactions with Dyneins in *Chlamydomonas* Flagella. MBoC.

[27] Bayly P.V., Lewis B.L., Ranz E.C., Okamoto R.J., Pless R.B., Dutcher S.K. (2011). Propulsive Forces on the Flagellum during Locomotion of *Chlamydomonas reinhardtii*. Biophys. J..

[28] Lefebvre P.A., Dentler W., Witman G. (1995). Cilia and Flagella Chapter 1 Flagellar Amputation and Regeneration in *Chlamydomonas*. Methods in Cell Biology.

[29] Guasto J.S., Johnson K.A., Gollub J.P. (2010). Oscillatory Flows Induced by Microorganisms Swimming in Two Dimensions. Phys. Rev. Lett..

[30] Böddeker T.J., Karpitschka S., Kreis C.T., Magdelaine Q., Bäumchen O. (2020). Dynamic Force Measurements on Swimming *Chlamydomonas* Cells Using Micropipette Force Sensors. J. R. Soc. Interface.

[31] Wong Y.J., Zhu L., Teo W.S., Tan Y.W., Yang Y., Wang C., Chen H. (2011). Revisiting the Stöber Method: Inhomogeneity in Silica Shells. J. Am. Chem. Soc..

[32] Ge J., Yin Y. (2008). Magnetically Tunable Colloidal Photonic Structures in Alkanol Solutions. Adv. Mater..

[33] Biswal S.L., Gast A.P. (2004). Micromixing with Linked Chains of Paramagnetic Particles. Anal. Chem..

[34] Xie S., Wu L., Tong Z., Chen H.-E., Chen Z., Uchimoto T., Takagi T. (2018). Influence of Plastic Deformation and Fatigue Damage on Electromagnetic Properties of 304 Austenitic Stainless Steel. IEEE Trans. Magn..

[35] Long J.C., Merchant S.S. (2008). Photo-Oxidative Stress Impacts the Expression of Genes Encoding Iron Metabolism Components in *Chlamydomonas*. Photochem. Photobiol..

[36] Glaesener A.G., Merchant S.S., Blaby-Haas C.E. (2013). Iron Economy in *Chlamydomonas reinhardtii*. Front. Plant Sci..

[37] Eckhardt U., Buckhout T.J. (1998). Iron Assimilation in *Chlamydomonas reinhardtii* Involves Ferric Reduction and Is Similar to Strategy I Higher Plants. J. Exp. Bot..

[38] Gao Y., Beerens J., van Reenen A., Hulsen M.A., de Jong A.M., Prins M.W.J., den Toonder J.M.J. (2015). Strong Vortical Flows Generated by the Collective Motion of Magnetic Particle Chains Rotating in a Fluid Cell. Lab Chip.

[39] Lamb H. (1895). Hydrodynamics.

[40] Liu Q., Prosperetti A. (2010). Wall Effects on a Rotating Sphere. J. Fluid Mech..

[41] Yagi T., Minoura I., Fujiwara A., Saito R., Yasunaga T., Hirono M., Kamiya R. (2005). An Axonemal Dynein Particularly Important for Flagellar Movement at High Viscosity: Implications from a New *Chlamydomonas* Mutant Deficient in the Dynein Heavy Chain Gene DHC9. J. Biol. Chem..

[42] Minoura I., Kamiya R. (1995). Strikingly Different Propulsive Forces Generated by Different Dynein-Deficient Mutants in Viscous Media. Cell Motil. Cytoskelet..

[43] McCord R.P., Yukich J.N., Bernd K.K. (2005). Analysis of Force Generation during Flagellar Assembly through Optical Trapping of Free-Swimming *Chlamydomonas reinhardtii*. Cell Motil. Cytoskelet..

